# Validation of a Novel Electronic Device for Medication Adherence Monitoring of Ambulatory Patients

**DOI:** 10.3390/pharmacy7040155

**Published:** 2019-11-20

**Authors:** Isabelle Arnet, Jean-Pierre Rothen, Valerie Albert, Kurt E. Hersberger

**Affiliations:** Department of Pharmaceutical Sciences, Pharmaceutical Care Research Group, University of Basel, CH-4056 Basel, Switzerland; jp.rothen@unibas.ch (J.-P.R.); kurt.hersberger@unibas.ch (K.E.H.)

**Keywords:** medication adherence, drug labelling, Time4Med^TM^, smart card, polypharmacy

## Abstract

Several methods exist for measuring medication adherence. The Time4Med^TM^ device (Adherence Innovations, Hong Kong) is a small, electronic card to affix on medication packaging that records date and time of intakes when a button is pushed. We aimed to validate the device with an emphasis on polypharmacy. Twenty volunteers used Time4Med^TM^ devices with a virtual thrice daily intake over 14 days. Diary-recorded date and time were compared to electronically-stored events. Functionality, reliability and recovery for different stress conditions were calculated. User‘s acceptability was assessed with the System Usability Scale (SUS). Eleven elderly outpatients (mean age 80.2 ± 8.1 years) taking >3 medications daily used the device over 4 weeks. Volunteers logged 847 events. Functionality (100%), sensitivity (94.9%), specificity (99.4%) and recovery (100%) were high. Dropping the smart card and storing it in a refrigerator caused either the recording of false events or no recording at all. The mean SUS score was 82.6 (SD 14.8), demonstrating excellent acceptability. Satisfaction was very high for volunteers and patients, except for pushing the button. Time4Med^TM^ devices are highly accurate in recording, retaining and delivering electronic data of multiple medication intake. They are well accepted by elderly patients. They can be recommended in clinical studies and for practitioners who desire to elucidate adherence patterns of ambulatory patients.

## 1. Introduction

Several methods are available for measuring adherence to medication with their respective pros and cons [1]. As a gold standard for research—but not yet for clinical use—electronic monitoring represents the most accurate measurement. It generates a time-stamp with the precise timing of medication taking behavior and reveals a comprehensive figure of an individual’s day-to-day drug intake. The electronic measurement of adherence to oral solid forms is currently feasible with electronic pill bottle caps (such as MEMS^®^, Medication Event Monitoring System; Aardex Ltd., Sion, Switzerland) or blister packaging equipped with an e-label, a so called Smart Blister [2]. Both systems monitor the removal of one single or lead drug. For inhaled medication, electronic rings can be attached to compatible asthma devices (such as SmartInhaler®, Adherium Ltd., Auckland, New Zealand) for the monitoring of every actuation [3]. One frequently mentioned drawback to such monitoring systems is that tracking time and date does not necessarily correlate to actual medication administration, e.g., patients can open/close the medication bottle without taking the medication. However, a study demonstrated that a patient’s intake behavior is not altered by electronic monitoring, and supported the validity of electronically recorded data [4].

The above-mentioned electronic systems have drawbacks of a practical nature. Electronic caps require the transfer of the medication of interest into a bottle after deblistering from the original packaging, which might give rise to stability issues. Commercially available packaging exists in a plethora of sizes and forms that renders the development of general e-labels unaffordable. Finally, smart inhalers are expensive. Beside the price of the device (approximately £100), access to a software that enables real-time monitoring after upload of the data to a mobile or cloud-based application costs monthly fees [5]. In addition, for all systems, monitoring of multiple medication with the same device is not possible.

With the emergence of mHealth (mobile Health) based on an unprecedented use of smart phones and wireless data transmission, a plethora of innovative mobile applications (apps) have been developed [6], some with the purpose of improving treatment adherence. Unfortunately, the app market is currently poorly regulated [7] and adherence apps can be summarized as advanced medication reminder apps [8], where the patient is the supplier and interpreter of their own personal data. Due to security and privacy issues in most countries, sharing in the cloud of stored patient data with healthcare providers in view of a professional interpretation is currently rendered impossible [9].

Under these circumstances, we aimed at testing an innovative device named Time4Med^TM^ for the electronic monitoring of medication adherence that fulfils the main requirements such as small size, ease of use, poor intrusiveness and affordability (the costs of a card are between € 15 and € 40 depending on volume. This price includes the dedicated app and a tablet computer). In addition, it can be used with polypharmacy that is, when a patient needs to take several medications daily [10]. Moreover, protected data transfer and analysis of adherence through healthcare providers (pharmacist, nurse, and general practitioner) is given. We present in this article the validation study with testing of the objective dimension (i.e., functionality and reliability) and the subjective dimension (i.e., the users’ acceptance and satisfaction) in an experimental design. In addition, we present metrics to calculate adherence from recorded electronic data, including polypharmacy or several intake times.

## 2. Materials and Methods 

### 2.1. Time4Med^TM^ Design

**Figure 1:**
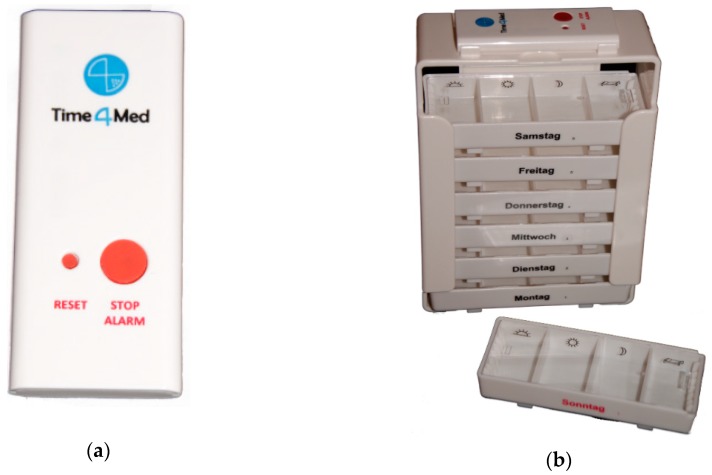
Time4Med^TM^ device (a) and affixed on the top of its corresponding commercially available pillbox (b). A clip permits to fix the smart card beneath each daily removable drawer.

**Figure 2:**
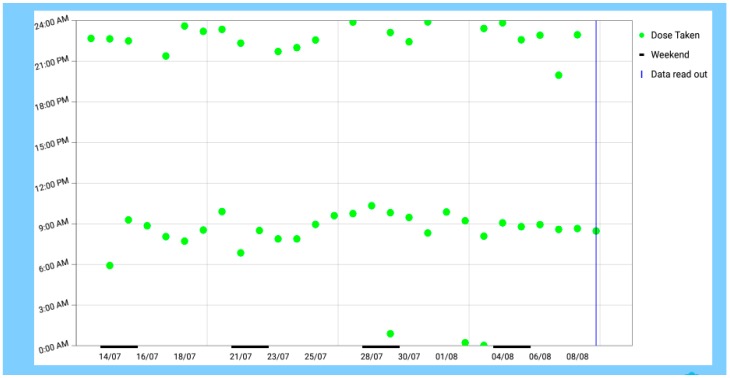
Scatter diagram of the intakes of a patient (green dots) with a two times a day regimen over 4 weeks displayed on a tablet computer with a dedicated app.

The small (75 × 32 × 7.4 mm) electronic, reusable device (Figure 1a; Adherence Innovations, Hong Kong) can be affixed to any medication packaging or clipped to its corresponding pillbox (Figure 1b), or affixed to any multicompartment adherence aid with double-sided adhesive tape. The Time4Med^TM^ device is composed of a Bluetooth Low Energy chip (BLE chip) which has a unique identification number in its memory, of a Near Field Communication chip (NFC chip), and of a battery. A time-stamp (date and time) is recorded on the BLE chip each time a patient pushes the red button (0.9 mm in diameter) for 3 seconds. A beeping sound is emitted, and data recording is blocked for the next 5 min to avoid multiple records should a patient push the button several times. Each time medication is taken, patients press the button. Several scenarios are possible for multiple medication (polypharmacy), depending on whether patients have the original packaging or pillboxes. Patients may press one button per medication when each packaging is equipped with a device. Alternatively, patients may press the same button several times according to the intake times when using pillboxes in which multiple medication was repackaged. To read data, a connection with the dedicated app on a tablet computer is established by holding the Time4Med^TM^ device (smart card) within 10 cm of the NFC antenna. The Bluetooth connection is activated automatically. The stored data are directly transferred to the dedicated app without intermediate storage on a server. Data can be downloaded from the tablet computer in csv format or displayed as scatter diagram within the dedicated app (Figure 2). Before the smart card is handed out to register data, a connection with the dedicated app is needed to synchronize date and time on the BLE chip.

### 2.2. Validation Study

We tested the Time4Med^TM^ devices regarding their technical performance (objective dimension, i.e., reliability) and the patient perspective (subjective dimension, i.e., usability, satisfaction, and acceptability) [11]. For the subjective dimension, we recruited elderly subjects between March and August 2018. They were living independently, self-managed their >3 medications (polypharmacy), and accepted to give insight into their medication intake. Interviews were performed at the patients’ home before and after a four-week use of the smart card. The instruction was “*Every time you take medication (one or several), please push the red button on the Time4Med^TM^ device for 3 seconds or until a beep sound is emitted*”. This observational study was approved by the ethics committee of Northwestern and Central Switzerland (EKNZ Nr. 2018–75).

For the objective dimension, we followed published data on the validation of Smart Blister [12] with a purposive sample of non-medicating volunteers to simulate routine use, and stress tests under laboratory conditions. We recruited 20 volunteers amongst students and PhD students affiliated to our research group between 1 and 28 February 2018. Volunteers were required to press the button for the study duration with the instruction of use as follows: “*Confirm a virtual treatment 3 times daily by pushing the red button on the Time4Med^TM^ device for 3 seconds or until a beep sound is emitted*”. Simultaneously, volunteers recorded the date and time of each simulated intake on a log sheet (index time) according to a personal clock. The stored data were read from the smart cards and compared to the index time.

**Figure 3:**
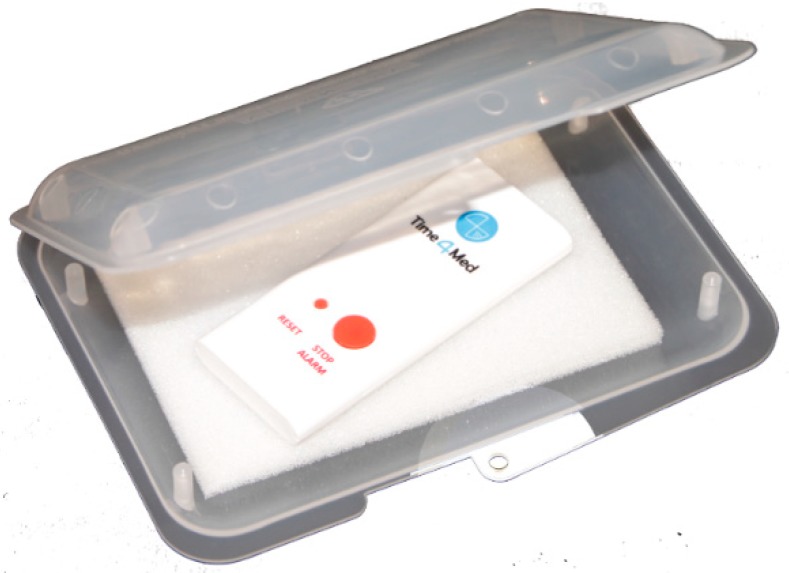
Smart card in the open plastic protective case.

The Time4Med^TM^ cards were exposed to the following conditions:

- Field: 20 smart cards were used at home by non-medicating volunteers.
- Purse: 2 smart cards were carried in women personal purses, without further protection.
- Dropped: 20 smart cards were used, then dropped from a height of 1.2 m once, and then used again once.
- Post mail: 2 smart cards were packed in a plastic protective case (135 × 99 ×18 mm; Renfer GmbH, Lengnau, Switzerland; see Figure 3), put into a common A5 envelope and post mailed.
- Refrigerator: 20 smart cards were stored in a common refrigerator at 2–12 °C.

We evaluated the following criteria. We defined the functionality of the smart cards as the percentage of readable devices after a period of use. Smart cards that could not be activated by the reader at the beginning of the study were excluded. The reliability of an electronic system, i.e., the degree of conformity of a recorded event to actual use [11], is estimated from its accuracy which is judged by its sensitivity and specificity values [13]. Sensitivity is defined as the proportion of events that were stored and indexed (true positive rate) [13] and was calculated by the formula (Number of electronically-stored events within +/− 5 min of index time/Number of diary-recorded events) × 100. The grace period of 5 min permits to eliminate the artificial time differences between the volunteers’ personal watches and the electronic clock of the devices. Specificity is defined as the proportion of events that were not stored and not recorded (true negative rate) [13] and was calculated by the formula 100 − [(Supernumerary electronically-stored events/Number of diary-recorded events) × 100].

We defined recovery as the percentage of identical events read out before and after a stress condition (i.e., dropping and post mail) and was calculated with the formula (Number of electronically-stored events after the stress condition/Number of electronically-stored events before the stress condition) × 100.

In the absence of rigorously validated measures of the acceptability of monitoring devices, we assessed acceptability with 5 questions informed through issues on ease of use, alarm and device size: [14] i) The smart card is easy to use; ii) The button is easy to find; iii) Pushing the button is easy; iv) The alarm is well audible; v) The size of the smart card is adequate. Volunteers and seniors answered on a 4-point Likert scale (from 1 = fully disagree to 4 = fully agree) after having used the smart card. A higher score (maximum of 4) indicates a more positive experience. The participants were asked to give comments in case of giving the marks 1 or 2.

Usability was assessed with the System Usability Scale (SUS) [15]. It is a reliable, valid, quick and low-cost questionnaire to measure the usability of computer systems such as software, mobile devices, websites and applications. It exists in several languages, including German [16] (see Appendix A). The participants answered the 10 statements on a 5-point Likert scale (from 0 = fully disagree to 4 = fully agree). For the scoring, half of the scores are reversed and the original scores of 0–40 are converted to 0–100. A SUS score >68 is considered above average [17], with a good acceptability for scores up to 73; excellent up to 85 and best imaginable up to 100.

## 3. Results

### 3.1. Field Condition

Twenty volunteers (9 students, 8 PhD students, 1 professor in pharmacy, 1 pharmacist, and 1 nurse) used a Time4Med^TM^ smart card over 14 consecutive days. The 20 smart cards were readable (functionality 100%). The volunteers recorded in their diaries 847 index times (average: 42.4, range: 40–47). A total of 812 events were electronically-stored (95.9%), 804 of them within 5 min of the index time (sensitivity 94.9%; Table 1). Five events from 3 smart cards were electronically-stored outside the index time (specificity 99.4%; Table 1). A total of 44 events were not electronically-stored although logged (5.2% false negative rate), 25 of them concerned the first 25 events of the same smart card and were due to unexplained technical failure. From another smart card, 18 events had a 335 min shift compared to the index time after the smart card was heavily dropped in a staircase from approximately 2.5 m. No additional events were stored around the time of connecting the smart cards with the tablet computer or reading the data. During the following 3 month period in which the smart cards were not used, no additional events were electronically-stored (100% accuracy in retaining stored electronic data). 

### 3.2. Purse

From the 83 times recorded in diaries, all were electronically-stored (sensitivity 100%). Two additional events were electronically-stored outside the index time (specificity 97.6%; Table 1).

### 3.3. Dropped

The 827 electronically events stored on the 20 smart cards were identical before and after dropping (recovery 100%). The button was then pushed once on each card. From the 20 new events, one had a 15 min shift compared to the index time (sensitivity 95.0%; Table 1).

### 3.4. Post Mail

Two smart cards that contained 11 electronically-stored events were post mailed to addresses in Switzerland and Germany on 28 January 2018. All envelopes arrived intact at destination within 2 days. All 22 electronically-stored events were identical before and after being post mailed (recovery 100%; Table 1)

### 3.5. Refrigerator

Each smart card was used 1 to 4 times daily on working days (Monday–Friday) between 29 January and 9 February 2018 (16 days). From the 560 diary-recorded events, 533 were electronically-stored, 518 of them within 5 min of the index time (sensitivity 92.5%). For three smart cards, a total of 15 events were electronically-stored outside the index time (specificity 97.3%; Table 1) and 27 events were missing. These three smart cards stopped recording after 3 and 9 days, respectively.

### 3.6. Summertime Conditions

The cards have been used in a study [18] in Switzerland with a temperate climate. There was no dysfunctioning over the summertime (June–September 2018) due to heat or humidity.

### 3.7. Acceptability

The seniors consisted of six women and five men, they were on average 80.2 ± 8.1 years old (range 65–95 years), lived alone (eight) or with a spouse (three), and had a number of daily medications ranging from four (four patients) to more than 10 (two patients). The visualizing graph of the daily intakes on the tablet computer was perceived as reinforcement to persevere when the profile was regular, or as incentive to improve when the profile was disparate. The five qualifiers “Ease of use”, “Finding the button”, “Pushing the button”, “Hearing the alarm” and “Size of the card” were judged equally satisfactorily by the volunteers (mean 3.3 ± 0.9) and the patients (mean 3.6 ± 0.7). “Pushing the button” was the most incriminated qualifier (mean 2.3 ± 0.9 (volunteers) and 3.3 ± 0.8 (patients)). They criticized the length of time needed to push the button (3 seconds), the lack of resistance or click tone to confirm the pushing, and the delay between pushing the button and the emission of the beep tone. Volunteers remarked that both pressure and dexterity were needed to push the button which might be challenging for elderly persons. This was confirmed by two patients who needed a lead pencil to push the button. The patients criticized also the size, which was somewhat too small (mean 3.5 ± 0.7). 

### 3.8. Usability

The mean SUS score was 83.6 ± 14.8 and indicates a usability between good and excellent.

### 3.9. Metrics

Transforming electronic monitoring information into one adherence value represents a challenge [19], especially with polypharmacy. Most measures present summary scores over the number of intakes each day, generally named “Taking adherence” or “Dosing adherence”. They have been calculated as (i) percent prescribed number of doses taken [20], (ii) percent of days with correct number of doses taken [20], or (iii) percent of prescribed doses taken on schedule (i.e., twice daily) [20], and ignore dose timing. For multiple regimens, the values obtained for each single medication are generally averaged [21]. 

When accounting for the precise time of the dosing events, measures of “Timing adherence” have been calculated, such as (iv) percent doses taken within a grace period of time (i.e., within 6 hours) [22]; (v) percent doses taken within a pre-set interval (e.g., 8–12 h apart) [23]; or (vi) Area Under the Curve on the basis of correct interval lengths between doses [24]. In all cases, the exact moment of the intake can be estimated as mean or median intake hour [25].

## 4. Discussion

We tested the performance of the new electronic device Time4Med^TM^ smart card in 20 healthy volunteers and 11 elderly outpatients with polypharmacy. In our field conditions that mimic daily use, functionality was 100% and reliability was at least 95% for all criteria. Under real-life conditions, acceptability and satisfaction were very high for all participants. These results are similar to other electronic monitors of commercially available blisters [26] or pressurized metered-dose inhalers [27]. However, we evaluated objective criteria (such as reliability and validity) as well as subjective criteria (such as users’ satisfaction and acceptability). Because electronic devices are increasingly promoted [28] to monitor the medication intake of individuals at their homes and detect medication-related problems, testing under real-life conditions is a prerequisite to application of the devices in clinical studies or daily practice.

The elevated rate of 5.2% false negative events (i.e., diary-recorded but not electronically-stored events) is misleading because it concerned only two smart cards. A heavy dropping was identified as the cause of the missing registration in one smart card, and another smart card lacked to record 25 events. Each card was tested prior to dispensing to patients that is, the button was pushed once and the device was read straight after, so that patients obtained well-functioning devices. However, this procedure cannot eliminate technical failure occurring during use. Real-time monitoring would avoid faulty device and loss of data [29]. In our field study, if we disregard the explainable missing events, that is, the faulty device and the heavy dropping, only one event was a false positive (0.1%). This remarkable result demonstrates the stability of the electronic recording when used appropriately.

The Time4Med^TM^ smart cards can pick up extraneous recordings when dropped or transported unprotected in a purse. However, when protected by a rigid plastic box, the smart card withstood national and international post mails. Consequently, usual precaution is recommended with the Time4med^TM^ as with any electronic device. Thus, when a patient returns their smart card and the recording of the events stopped abruptly although the patients assure that they have pushed the button regularly, elucidating a mishandling of the device might be a logical step, followed by the patient education on correct storage of the device. The Time4Med^TM^ smart card is vulnerable after 2 days in the refrigerator, probably due to the condensation that may seep into the device and short cut the circuits. This is inherent to any electronic device. Consequently, the Time4Med^TM^ smart card should not be attached to medications that need to be stored at cold temperature. However, if attached on the refrigerator door for example, the smart card can well serve to monitor the intake of a refrigerated medication, in addition to other medications stored at room temperature.

We have selected a grace period of 5 min between index time and electronically-stored events which results in accepting a difference of <6 min between a diary-recorded and an electronically-stored event. This is comparable to the 10 min that have been used in a similar study [12]. By doing this, we have avoided misclassification when minor differences existed between a personal watch and a radio-controlled clock, and we have allowed some spare time between the moment the volunteers have activated the smart card and the moment they have filled in the diary. It is conceivable that smart cards and log sheets were not stored at the same place. If the grace period is too small, some electronic data will erroneously be categorized as supernumerary electronically-stored and thus, increase the rate of false positive values. However, in daily practice, an even greater lag time between the intake of the medication and the recording on the device might be negligible, considering that the interpretation of adherence data is most sensitive to dose omission and treatment stop [30]. One exception may be events close to midnight that would theoretically be counted to the next day. This is the reason why a “day” starts at 3am for adherence calculations.

We did not explicitly assess obtrusiveness which is defined as a technology that is perceived as undesirable because it is physically and/or psychologically prominent [31]. However, neither the volunteers nor the patients made any comment in this sense. On the contrary, the patients wished a somewhat larger device. Thus, we consider that the Time4Med^TM^ smart card is not obtrusive. Further, the smart card was well accepted. Monitoring ones’ intakes was not a concern for the patients and this degree of supervision was admitted. The graphical representation of the daily intakes on the tablet computer was well accepted. This is consistent with other articles where graphs of electronic monitoring were a means of motivation [32]. Finally, even if the smart card (as every indirect assessment method) cannot confirm the ingestion of the medications, the visualization helped single patients to detect irregular or deleterious medication intake behaviors, and to correct them with the support of a pharmacist. Thus, looking at one’s graph acted as a motivator to take one’s medication and reinforced adherence. 

Thinking about the elderly, volunteers questioned the beeping tone and the pushing of the button as being problematic for the elderly, that is, not enough loud and too difficult to push. These assumptions were partly confirmed by the elderly patients. To our surprise, they did not criticize the sound, but solely the pushing of the button and developed strategies in case of hindrance such as the use of a pencil as pushing help. Some volunteers were also doubtful about the size of the button, which could be difficult to find with poor eyesight, and there was potential for confusion between the red button and the blue logo, both of similar sizes. However, the patients had no difficulties finding the button, probably due to its rubber surface and its slight depression in the plastic case. Thus, tactile sense seems sufficient to find the red button on the smart card. Nevertheless, we recommend to demonstrate the functioning of the smart card by asking the patient to push the button once in front of the healthcare provider. This event must be recorded and excluded from the interpretation of adherence data. In addition, a leaflet with a clear description of the smart card and its purpose could help the patient and their careers remember all information. 

The smart card must be returned to the healthcare providers for the extraction of the data. Data protection and privacy are becoming increasingly restricted through laws governing electronic devices. Thus, the non-remote but near field technology transmission of patients’ data from the Time4Med^TM^ smart card to a personal tablet computer that belongs to a healthcare provider represents a highly secured method. Further, the synchronization of the device clock with the local time on the tablet computer is performed automatically during data reading. However, a country’s daylight savings period is not taken into consideration in the smart card memory. Thus, an eventual time discrepancy of 1 hour needs to be considered when interpreting the data around the end of March and October.

Battery depletion of the smart card can be checked at any time. The manufacturer guarantees 3 years after up to 6 intakes daily with once monthly data reading. Thus, if used for a time period of 1 month (to assess baseline intake behavior) followed by 3 more months after an adherence intervention (to assess changes in intake behavior), the same smart card can be re-used for 12 patients, provided that the data have been deleted (this feature exists) before handing out the smart card to the next patient. We recommend exchanging the smart card monthly to permit a maximal data loss of 4 weeks. As costs for electronic devices reduce and tablet computers’ interfaces become uncomplicated for practitioners and patients, the Time4Med^TM^ smart card represents a long-awaited device to improve adherence, including to polypharmacy, in outpatients as healthcare provider can immediately interpret patient data and provide a tailored pharmaceutical counselling. In addition, the Time4Med^TM^ smart card can assess adherence in clinical trials from control and intervention patients equally, and enables comparison of the data.

Time4Med^TM^ can be used in practice to monitor adherence to multiple medication regimen even if it cannot distinguish which medication has been taken at a given time. Further research is now needed to determine cut-off values that can distinguish acceptable from unacceptable intake behaviors, taking the pharmacological profile of the medication into account [33].

Our study has some strengths. First, we tested the technical performance and the usability of the device with healthy non-medicating volunteers and polymedicated elderly patients (65–95 years) who represent the target population. Elderly patients typically store medications in multi-compartment pillboxes [34]. Second, even if the device only registers medication intake with a push on the button but does not register per medication, the information of several consecutive intakes per day is valid because with multiple medication regimen, patients generally take several pills together according to the treatment plan. In addition, the Time4Med^TM^ device can be used when patients use multicompartment pillboxes to manage their medications.

We acknowledge some limitations. First, the SUS was developed to measure more complex systems than testing the usability of pushing a button. Nevertheless, in absence of other validated tests in this field, we consider our results as unbiased compared to a corresponding yes/no question. Second, we discussed battery depletion but did not conduct a test to validate long-term use of the device (e.g., several months). Third, we are not aware of other similar currently available products. Thus, we cannot compare Time4Med^TM^ and further highlight its unique properties. Fourth, we performed short-term monitoring over one month. Thus, we cannot exclude that people will stop practicing the behavior of pressing the button in the long run. However, preliminary data from an ongoing study [18] indicate that monitoring over 12 months is well accepted. Finally, the device itself is unable to tell which medication a patient took if prescribed several, nor it can detect whether a patient took one or several medications. This is inherent in every electronic system that monitors intake indirectly. However, Time4Med^TM^ enables to detect deviant behavior and especially omitted doses, whose corresponding medication can be unveiled during a consultation.

## 5. Conclusions

The Time4Med^TM^ devices are highly accurate in recording, retaining and delivering electronic monitoring adherence data. They are easy to use and well accepted by elderly patients. The small cards can be recommended for practitioners who desire to elucidate adherence behaviors of ambulatory patients, and also with polypharmacy and in clinical trials. Further research is now needed to help practitioners interpret the recorded data and ultimately support clinical decision making.

## Figures and Tables

**Table 1:** Detailed results of the tests run by volunteers (field condition) and different stress conditions with the Time4Med^TM^ smart cards. Refer to the text for the definitions of sensitivity, specificity and recovery.

	Conditions [Number of Smart Cards]					
Events (Number)	Field [n = 20]	Purse [n = 2]	Dropped (Recorded before Dropping) [n = 20]	Dropped (Recorded after Dropping) [n = 20]	Post Mail [n = 2]	Refrigerator [n = 20]
diary-recorded	847	83	827	20	22	560
electronically-stored	812	85	827	20	22	533
electronically-stored +/− 5 min	804	83	827	19	22	518
supernumerary electronically-stored (false positive)	5	2	0	0	0	15
not electronically-stored although diary-recorded (false negative)	44	0	0	0	0	27
Sensitivity	94.9%	100%	-	95%	-	92.5%
Specificity	99.4%	97.6%	-	-	-	97.3%
Recovery	-	-	100%	-	100%	-

## Appendix A

**Table A1:** Ten questions of the System Usability (SUS) in English (left) and German (right).

System Usability Scale	Fragebogen zur System-Gebrauchstauglichkeit
I think that I would like to use this system frequently.	Ich denke, dass ich das System gerne häufig benutzen würde.
I found the system unnecessarily complex.	Ich fand das System unnötig komplex.
I thought the system was easy to use.	Ich fand das System einfach zu benutzen.
I think that I would need the support of a technical person to be able to use this system.	Ich glaube, ich würde die Hilfe einer technisch versierten Person benötigen, um das System benutzen zu können.
I found the various functions in this system were well integrated.	Ich fand, die verschiedenen Funktionen in diesem System waren gut integriert.
I thought there was too much inconsistency in this system.	Ich denke, das System enthielt zu viele Inkonsistenzen.
I would imagine that most people would learn to use this system very quickly.	Ich kann mir vorstellen, dass die meisten Menschen den Umgang mit diesem System sehr schnell lernen.
I found the system very cumbersome to use.	Ich fand das System sehr umständlich zu nutzen.
I felt very confident using the system.	Ich fühlte mich bei der Benutzung des Systems sehr sicher.
I needed to learn a lot of things before I could get going with this system.	Ich musste eine Menge lernen, bevor ich anfangen konnte das System zu verwenden.

## References

[1] Osterberg L., Blaschke T. (2005). Adherence to medication. N. Engl. J. Med..

[2] van Onzenoort H.A., Neef C., Verberk W.W., van Iperen H.P., de Leeuw P.W., van der Kuy P.-H.M. (2012). Determining the feasibility of objective adherence measurement with blister packaging smart technology. Am. J. Health Syst. Pharm..

[3] Gregoriano C., Dieterle T., Dürr S., Arnet I., Hersberger K.E., Leuppi J.D. (2017). Impact of an electronic monitoring intervention to improve adherence to inhaled medication in patients with asthma and chronic obstructive pulmonary disease: Study protocol for a randomized controlled trial. JMIR Res. Protoc..

[4] Wagner G.J., Ghosh-Dastidar B. (2002). Electronic monitoring: Adherence assessment or intervention?. HIV Clin. Trials.

[5] National Institute for Heath and Care Excellence NICE (2017). Smartinhaler for Asthma—Medtech Innovation Briefing. https://www.nice.org.uk/advice/mib90/resources/smartinhaler-for-asthma-pdf-63499461673669.

[6] World Health Organization WHO (2017). mHealth—New Horizons for Health through Mobile Technologies. WHO Library Cataloguing-in-Publication Data. http://www.who.int/goe/publications/goe_mhealth_web.pdf.

[7] Food and Drug Administration FDA (2015). Mobile Medical Applications—Guidance for Industry Food Drug Administration Staff. http://www.fda.gov/downloads/MedicalDevices/DeviceRegulationandGuidance/GuidanceDocuments/UCM263366.pdf.

[8] Santo K., Richtering S.S., Chalmers J., Thiagalingam A., Chow C.K., Redfern J. (2016). Mobile phone apps to improve medication adherence: A systematic stepwise process to identify high-quality apps. JMIR mHealth uHealth.

[9] Dehling T., Gao F., Schneider S., Sunyaev A. (2015). Exploring the far side of mobile health: Information security and privacy of mobile health apps on iOS and Android. JMIR mHealth uHealth.

[10] Hughes C.M., Cadogan C.A., Patton D., Ryan C.A. (2016). Pharmaceutical strategies towards optimising polypharmacy in older people. Int. J. Pharm..

[11] De Bleser L., De Geest S., Vincke B., Ruppar T., Vanhaecke J., Dobbels F. (2011). How to test electronic adherence monitoring devices for use in daily life: A conceptual framework. Comput. Inform. Nurs..

[12] Jekle C., Krämer I. (2008). OtCM (Objective therapy Compliance Measurement): Smart blister packages for measuring patient compliance. Hosp. Pharm. Eur..

[13] Chu K. (1999). An introduction to sensitivity, specificity, predictive values and likelihood ratios. Emerg. Med..

[14] Chan A.H.Y., Stewart A.W., Harrison J., Black P.N., Mitchell E.A., Foster J.M. (2017). Electronic adherence monitoring device performance and patient acceptability: A randomized control trial. Expert Rev. Med. Devices.

[15] Brooke S. (2013). SUS—A retrospective. J. Usability Stud..

[16] Rummel B. (2015). System Usability Scale—jetzt auch auf Deutsch. https://experience.sap.com/files/System_Usability_Scale_A4_DE.doc.

[17] Bangor A., Kortum P., Miller J. (2009). Determining what individual SUS scores mean: Adding an adjective rating scale. J. Usability Stud..

[18] Polymeris A.A., Albert V., Hersberger K.E., Engelter S.T., Schaedelin S., Arnet I. (2018). Protocol for MAAESTRO: Electronic Monitoring and improvement of Adherence to direct oral Anticoagulant treatment-a randomized crossover study of an Educational and reminder-based intervention in ischemic STROke patients under polypharmacy. Front. Neurol..

[19] Hartman L., Lems W.F., Boers M. (2019). Outcome measures for adherence data from a medication event monitoring system: A literature review. J. Clin. Pharm. Ther..

[20] Herzer M., Ramey C., Rohan J., Cortina S. (2012). Incorporating electronic monitoring feedback into clinical care: A novel and promising adherence promotion approach. Clin. Child. Psychol. Psy..

[21] van Bruggen R., Gorter K., Stolk R.P., Zuithoff P., Klungel O.H., Rutten G.E.H.M. (2009). Refill adherence and polypharmacy among patients with type 2 diabetes in general practice. Pharmacoepidemiol. Drug Saf..

[22] Zeller A., Schroeder K., Peters T.J. (2008). An adherence self-report questionnaire facilitated the differentiation between nonadherence and nonresponse to antihypertensive treatment. J. Clin. Epidemiol..

[23] Schmitz J.M., Sayre S.L., Stotts A.L., Rothfleisch J., Mooney M.E. (2005). Medication compliance during a smoking cessation clinical trial: A brief intervention using MEMS feedback. J. Behav. Med..

[24] Sulaiman I., Seheult J., MacHale E., Boland F., O’Dwyer S.M., Rapcan V. (2016). A method to calculate adherence to inhaled therapy that reflects the changes in clinical features of asthma. Ann. Am. Thorac. Soc..

[25] Arnet I., Walter P.N., Hersberger K.E. (2013). Polymedication Electronic Monitoring System (POEMS)—A new technology for measuring adherence. Front. Pharm..

[26] De Bleser L., Vincke B., Dobbels F. (2010). A new electronic monitoring device to measure medication adherence: Usability of the Helping Hand™. Sensors.

[27] Foster J.M., Smith L., Usherwood T., Sawyer S.M., Rand C.S., Reddel H.K. (2012). The reliability and patient acceptability of the SmartTrack device: A new electronic monitor and reminder device for metered dose inhalers. J. Asthma.

[28] Shtrichman R., Conrad S., Schimo K., Shachar R., Machluf E., Mindal E. (2018). Use of a digital medication management system for effective assessment and enhancement of patient adherence to therapy (ReX): Feasibility study. JMIR Hum. Factors.

[29] Bionghi N., Daftary A., Maharaj B., Msibi Z., Amico K.R., Friedland G. (2018). Pilot evaluation of a second-generation electronic pill box for adherence to Bedaquiline and antiretroviral therapy in drug-resistant TB/HIV co-infected patients in KwaZulu-Natal, South Africa. BMC Infect. Dis..

[30] Blaschke T.F., Osterberg L., Vrijens B., Urquhart J. (2012). Adherence to medications: Insights arising from studies on the unreliable link between prescribed and actual drug dosing histories. Ann. Rev. Pharm. Toxicol..

[31] Hensel B.K., Demiris G., Courtney K.L. (2006). Defining obtrusiveness in home telehealth technologies. J. Am. Med. Inform. Assoc..

[32] Haberer J., Kahane J., Kigozi I., Emenyonu N., Hunt P., Martin J. (2010). Real-time adherence monitoring for HIV antiretroviral therapy. AIDS Behav..

[33] Burnier M. (2019). Is there a threshold for medication adherence? Lessons learnt from electronic monitoring of drug adherence. Front. Pharm..

[34] Gould O.N., Todd L., Irvine-Meek J. (2009). Adherence devices in a community sample: How are pillboxes used?. Can. Pharm. J..

